# Higher Family Affluence is Associated With Multi-Sport Participation Among Irish Youth

**DOI:** 10.1177/00315125231185653

**Published:** 2023-06-27

**Authors:** Philip E. Kearney, Ian Sherwin, Wesley O’Brien, Alan M. Nevill, Kwok Ng

**Affiliations:** 1 Department of Physical Education and Sports Sciences, 150229 University of Limerick, Limerick, Ireland; 2 Sport and Human Performance Research Centre, 150229 University of Limerick, Limerick, Ireland; 3 Movement & Skill Acquisition Ireland, Cork, Ireland; 4 School of Education, Sports Studies and Physical Education, 8795 University College Cork, Cork, Ireland; 5 Faculty of Education, Health & Wellbeing, 8695 University of Wolverhampton, Wolverhampton, UK; 6 Faculty of Education, University of Turku, Rauma, Finland; 7 School of Educational Sciences and Psychology, University of Eastern Finland, Joensuu, Finland

**Keywords:** early sport specialization, socioeconomic status, sampling, multi-sport youth

## Abstract

The impact of early single sport participation among young people has received much attention, with both sport leaders and pediatricians advocating multi-sport participation at least until early adolescence. In this study we explored the association between family socioeconomic status and level of Irish youth specialization in sport. We relied on data from the Children’s Sport Participation and Physical Activity (CSPPA) study, involving a representative sample of 3499 Irish children and adolescents aged 10–15 years. We analyzed data from questions related to the number of sports played, the number of days per week the youth were engaged in sport, and family affluence (as a proxy measure for socioeconomic status). Youth sport specialization before 12 years of age (males 5.7%; females 4.2%) and even between 13–15 years of age (males, 7.8%; females, 5.8%) was uncommon. However, lower levels of specialization were associated with higher socioeconomic status in that more children with high family affluence participated in multiple sports. Careful consideration should be given to whether low socioeconomic status may act as a barrier to participation in multiple sports.

## Introduction

Youth sport specialization refers to young athletes focusing their efforts upon participation in a single sport and adopting adult-like training practices, with the aim of achieving personal and/or elite-referenced high-performance levels (Brenner, 2016). Specialization is a natural part of many young peoples’ journeys through sport (Côté & Vierimaa, 2014; MacPhail et al., 2010), although the age and manner in which specialization occurs is highly variable (Huxley et al., 2017; Storm et al., 2012). *Early* specialization refers to a child’s engagement in extensive and intensive adult-like sports training before they are physically ready to do so; this early specialization is generally assumed to be prior to the age of 12 years (Downing et al., 2022; Jayanthi et al., 2020; Mosher et al., 2020). In recent years, the impact of young people specializing prematurely in a single sport has received much attention (DiSanti & Erickson, 2021; Mosher et al., 2020; Zoellner et al., 2021), with different professional groups advocating for multi-sport participation until at least early adolescence (e.g., International Olympic Committee, Bergeron et al., 2015; American Academy of Pediatrics, Brenner, 2016).

While there appears to be a popular belief that early specialization is associated with an increased likelihood of attaining sporting success as an adult (Post et al., 2019, Post et al., 2021b), retrospective analyses of the developmental pathways of expert sports performers have repeatedly found that early specialization is not a pre-requisite to reaching high-performance sport capabilities as an adult (for reviews, see DiSanti & Erickson, 2021; Kliethermes et al., 2020). For example, less than one-fifth of collegiate athletes in the United States specialized before age 15 years (Rugg et al., 2021), and only 44.5% of surveyed professional athletes had specialized in playing a single sport during their youth, with these athletes having begun this specialization at an average age of 14.09 years (*SD* = 2.79) (Buckley et al., 2020). Not only has past research found no substantial benefit to early specialization, there is some suggestion that early specialization is associated with increased risk of overuse injuries (Bell et al., 2018a; Biese et al., 2020; Carder et al., 2020) and maladaptive psycho-social outcomes, such as burnout, sport devaluation, and psychological need dissatisfaction (Giusti et al., 2020; McFadden et al., 2016; Rugg et al., 2021; Waldron et al., 2019). While these negative outcomes have not always been identified (Downing et al., 2022; Patel & Jayanthi, 2018), the current research consensus is that early sport specialization is not required to achieve success at elite adult levels (Goodway & Robinson, 2015; Kliethermes et al., 2020).

Establishing the prevalence of early specialization is an important means of guiding administrators and sport development leaders in their allocation of resources for educational initiatives. Most current research on levels of early specialization in sport has been based on populations from the United States of America (e.g., Biese et al., 2020; Brooks et al., 2018; Field et al., 2019; Kliethermes et al., 2018; McLeod et al., 2019; Post et al., 2017, 2020, 2021a). Within this research, the predominant definition of specialization has been that of Jayanthi (Jayanthi et al., 2015, 2020) who suggested that highly specialized youth are those who can identify a primary sport, have quit other sports to focus on this sport, and who train more than eight months of the year in this sport. McLeod et al. (2019) reported that 25.5% of 11–12 year olds participating in soccer leagues were highly specialized; and, amongst 13–14 year olds, that figure increased to 36.5%. A cross-sectional study of 12 year olds participating within summer athletic tournaments reported that 30.2% were highly specialized and that figure gradually increased from 38.1% to 47.4% from age 13–15 years old (Post et al., 2017). Emerging data has also revealed similar specialization rates outside of the United States (McGowan et al., 2020; Nagano & Oyama, 2023; Whatman et al., 2021; Zoellner et al., 2022). For example, Zoellner et al. (2022) reported that 35% of a sample of New Zealand football players aged 10–13 years of age were highly specialized, increasing to 49% for children aged 13–15 years. Using the same survey, McGowan et al. (2020) reported that 20% of 10–13 year old New Zealand children attending a national sports event were categorized as highly specialized. Thus, early specialization appears to be a significant issue in multiple countries.

Numerous factors influence the degree of sport specialization within a population, including variation in the age range of participants, school sizes (Bell et al., 2016), urban versus rural locations (Bell et al., 2018b) and the specific sports played (Pasulka et al., 2017). Of particular note, a consistent recent finding within the United States has been that children of a higher socioeconomic status were more likely to specialize (Jayanthi et al., 2018; Post et al., 2018, 2019, 2021b). For example, a survey of 949 parents of youth athletes between 10–18 years old found that, as total household income increased, children were more likely to be classified as highly specialized (Post et al., 2018). It is also conceivable that low socioeconomic status may act as a barrier to multi-sport participation (Badura et al., 2021; Baker et al., 2021; Owen et al., 2022). In cultures outside the United States, early specialization may result from a paucity of available sport choices, rather than a deliberate decision by the athlete and his/her parents/coaches to specialize (Green & Smith, 2016). Such an interpretation is supported by the most recent systematic review of the evidence for socioeconomic disparities in sport participation, which concluded that children and adolescents living in higher socioeconomic status households were more likely to participate in sports than their peers of lower socioeconomic status (Owen et al., 2022).

Limited research has examined the role of socioeconomic status on sport specialization outside of the United States. One exception is that of Winn et al. (2017) who examined the sport participation profile of 590 under-15 rugby players selected for regional representative sides within Wales. Players from the most economically deprived population quintile (as indexed by the Welsh Index of Multiple Deprivation) had engaged in significantly fewer other sports than players in the three least economically deprived quintiles. The consistent association between high socioeconomic status and specialization amongst populations based in the United States (Jayanthi et al., 2018; Post et al., 2018, 2019, 2021b), coupled with the finding that individuals from low socioeconomic status backgrounds typically participate in less sport than their more affluent peers (Owen et al., 2022; Winn et al., 2017), suggest that an investigation of the relationship between socioeconomic status and sport specialization is required in a variety of populations outside of the United States.

A country’s national youth sport culture is determined by factors such as the importance placed on sport, the extent of adult involvement (i.e., the prevalence of supervised sport vs. free play), the purpose of sport (e.g., winning vs. holistic development), how sport is organized (e.g., an emphasis on single sports or multi-sports, traditional seasons for sports, sports organized through school or club settings), and the extent to which adolescents have a voice in sport (Green & Smith, 2016; Hulteen et al., 2017; Waldahl & Skille, 2016). On the island of Ireland, the combination of the cultural importance of Gaelic Games and the amateur status of the high-performance game (Geary et al., 2022) mean that there may be fewer pressures to specialize within the Irish context than in countries like the United States. As such, our purposes in this study were to examine the level of early sport specialization within Ireland, and to determine whether it might be related to socioeconomic status. While Woods et al. (2019) demonstrated that Irish children from low socioeconomic backgrounds were less likely to participate in community sport than children from higher socioeconomic backgrounds, these investigators did not consider sport specialization.

## Method

### Research Design

In this study, we relied on data previously collected by Woods et al. (2019) in the Children’s Sport Participation and Physical Activity (CSPPA) study, which provided a cross-sectional perspective of participation in general physical activity, sports participation and physical education across the island of Ireland. Data were collected in classrooms through researcher-administered self-report surveys on tablets or computers during the spring semester 2018. All surveys were completed anonymously and voluntarily. Only respondents whose parents or guardians gave informed consent for their participation were included in the study, and respondents understood that their survey completion could be terminated at any time, including the right for withdrawal upon survey completion. The study received approval from an Institutional Ethical Review Board.

### Participant Sample

To produce a national representative sample, proportional sampling of schools was based on a school’s population gender (male, female or mixed), location (urban or rural), size (small, medium or large), and status of disadvantaged or non-disadvantaged students. In the Republic of Ireland, disadvantaged schools were those listed within the program for Delivering Equality of Opportunity in Schools. Within Northern Ireland, schools were classified on the basis of the percentage of students with free meals. Full details on sampling, data collection and data processing are available in previous CSPPA publications (Woods et al., 2019). The methods and results within this paper represent a secondary analysis of the data from CSPPA items related to specialization in sport and socioeconomic status. As the focus of our study was on early specialization, our data analysis was restricted to children and adolescents aged between 10–15 years who were actively participating in community sport (i.e., in a non-school sports club) at least once per week. The dataset from the Children’s Sport Participation and Physical Activity Study (CSPPA) is available for further analysis at https://www.ucd.ie/issda/data/csppa/csppa1718/. To facilitate researchers who may wish to re-examine our data, the specific steps by which we identified the subsample for this analysis are available from the corresponding author on request.

### Measures

Socio-demographic information was collected at the individual level and included age, gender, and socioeconomic status. The CSPPA utilized the Family Affluence Scale II (FAS (Boyce et al., 2006)) as a proxy indicator of socioeconomic status, whereby participants answered four questions related to the family’s material assets (number of cars, bathrooms, computers and recent foreign holidays). Consistent with Inchley et al. (2016), the top 20% of scores constituted high affluence, the bottom 20% constituted low affluence, and the remaining scores moderate affluence.

Questions asked within the CSPPA allowed an analysis of the level of sport specialization, defined on the basis of two variables: the number of clubs in which the participant was a member (1, 2, 3…7+) and the number of days per week that the participant was engaged in community sport (1, 2–3 or 4+ days per week) (Downing et al., 2020; Nagano & Oyama, 2023). Individuals were classified as ‘specialized’ if they participated 4+ days per week (the highest option on the CSPPA survey) in a single sport (4+DSS). Individuals were classified as 4+DMS if they participated 4+ days per week in multiple sports. Individuals were classified as 1-3DSS if they participated in a single sport 1–3 days per week. Finally, individuals were classified as 1-3DMS if they participated in multiple sports for a total of 1–3 days per week.

With Gaelic Games, children often participate within both Gaelic Football and Hurling/Camogie^1^ within the one club. While both games may be classified as invasion field sports, there are considerable differences between them (e.g., one is played with a large round ball akin to a soccer ball, whereas the other is played with a stick and small ball, similar to field or ice hockey). In addition, the coaches involved with football and hurling teams are often different. As such, we pre-processed the data such that any child who identified playing both football and hurling was counted as participating in multiple sports, even if they only identified being a member of a single club. *Early* specialization was defined as meeting the criteria for specializing at or before 12 years of age (Jayanthi et al., 2020; Mosher et al., 2020). Consequently, and in accordance with the Sampling and Specializing phases of the Developmental Model of Sport Participation (Côté, 1999; Côté et al., 2007), participants were classified into two age groups: 10–12 years of age, and 13–15 years of age.

### Data Analysis

Chi squared tests of Independence provided a measure of association between specialization status (4+DSS, 4+DMS, 1-3DSS or 1-3DMS) and family affluence (low, medium and high). We set alpha to .05 for determining statistical significance, adjusted in accordance with Holm-Bonferroni (Holm, 1979) to account for the multiple comparisons undertaken. To determine the source of the deviation from the expected distributions within the contingency tables, inspection of the percentage of cases in each category was supported by analysis of the standardized residuals (SR); a value of +1.96 indicated an overrepresentation within a category, while a value of −1.96 indicated an underrepresentation in a category. Cramer’s V provided a measure of effect size, with .045, .134 and .224 providing a measure of small, moderate and large effects, respectively, due to the six degrees of freedom of the association (Cohen, 1988). Consistent with Baker et al.’s (2021) call to examine the constituent elements of specialization, we used Ordinal Logistic Regression to further explore whether family affluence (low, medium and high) could be predicted by characteristics of sport participation (single vs. multi-sport participation; number of days per week playing sport: 1, 2–3, or 4+). Gender was entered into the model as a factor, while age was entered as a covariate.

## Results

### General Characteristics of Participation in Sport

Our final sample was comprised of 3,499 children aged 10–15 years who were actively participating in at least one community sports club at least 1 day per week. A breakdown of results regarding these participants’ sports engagement activities is provided in Table 1. Overall, early specialization was infrequent amongst children aged 10–12 years (boys, 5.7%; girls, 4.2%). Similarly, amongst adolescents aged 13–15 years, specialization continued to be infrequent (boys, 7.8%; girls, 5.8%). With the exception of boys aged 10–12 years from low affluence families, participation in multiple sports was more common than participation in a single sport. With the exceptions of boys from high affluence families aged 10–12 years and 13–15 years, participation in sport 1–3 days per week was more common than participation 4+ days per week.

**Table 1.** Relationships Between Family Affluence and Patterns of Sport Participation.

		Pattern of Sport Participation					χ^2^	*p*	V
		*n*	4+DSS	4+DMS	1-3DSS	1-3DMS			
Boys 10–12 y	Low FAS	107	6.5%	20.6%	45.8%	21.1%	36.9	<.001	.24
	Medium FAS	385	4.7%	37.1%	22.9%	35.3%			
	High FAS	152	7.9%	46.1%	23.0%	23.0%			
Girls 10–12 y	Low FAS	141	7.1%	26.2%	34.0%	32.6%	16.0	.014	.15
	Medium FAS	410	4.4%	28.5%	26.3%	40.7%			
	High FAS	189	1.6%	38.1%	24.3%	36.0%			
Boys 13–15 y	Low FAS	190	11.1%	29.5%	23.7%	35.8%	15.6	.016	.12
	Medium FAS	694	7.5%	36.0%	20.3%	36.2%			
	High FAS	218	6.0%	46.3%	18.3%	29.4%			
Girls 13–15 y	Low FAS	162	5.6%	24.7%	30.9%	38.9%	13.8	.032	.12
	Medium FAS	580	6.0%	30.5%	25.7%	37.8%			
	High FAS	271	5.5%	39.1%	19.2%	36.2%			

*Note*: FAS = Family Affluence Scale II; 4+DSS = participating 4+ days/week in a single sport (i.e., specialised); 4+DMS = participating 4+ days/week in multiple sports; 1-3DSS = participating 1–3 days/week in 1 club; 1-3DMS = participating 1–3 days/week in multiple sports. *n* = number of participants within each category. y = years. Rows do not always add up to 100% due to rounding. χ^2^ = Chi-Squared statistic, *p* = *p* value, and V = Cramer’s V effect size.

### Associations Between Family Affluence and Early Sport Specialization (Age 10–12 Years)

Amongst boys aged 10–12 years, a statistically significant association between family affluence and level of specialization was noted (χ^2^ = 36.9, *p* < .001, V = .24). Specifically, fewer children with low FAS were classified in the 4+DMS category (20.6%; SR = −2.73) compared to children with high FAS (46.1%; SR = 1.95) and children with moderate FAS (37.1%; SR = .21). Correspondingly, more children with low FAS were classified in the 1-3DSS category (45.8%; SR = 3.82) relative to children from the high FAS (23.0%; SR = −.87) and moderate FAS groups (22.9%; SR = −1.46).

Amongst girls aged 10–12 years, a statistically significant association between family affluence and level of specialization was noted (χ^2^ = 16.0, *p* = .014, V = .15). Consistent with the pattern revealed for the boys of this age group, fewer children of low FAS were categorized as 4+DMS (26.2%; SR = −.92) than children of high FAS (28.1%; SR = 1.88) and moderate FAS (28.5%; SR = −.73). More children of low FAS were categorized as 1-3DSS (34.0%; SR = 1.53) relative to children from the high FAS (24.3%; SR = −.79) or moderate FAS group (26.3%; SR = −.37).

### Associations Between Family Affluence and Sport Specialization (Age 13–15 Years)

At 13–15 years of age, the proportion of children classified as specialized remained small (boys, 7.8%; girls, 5.8%), and much lower than the proportion of young adolescents who were active in multiple sports 4+ days/week (boys, 36.9%; girls, 31.9%). Amongst boys aged 13–15 years, there was a statistically significant association between family affluence and level of specialization (χ^2^ = 15.6, *p* = .016, V = .12). Inspection of the standardized residuals revealed that more boys from a high FAS background were categorized as 4+DMS (46.3%; SR = 2.28) than expected, compared to boys from a moderate (36.0%; SR = −.39) or low (29.5%; SR = −1.69) FAS background. Amongst girls aged 13–15 years, there was a statistically significant association between family affluence and level of specialization; (χ^2^ = 13.8, *p* = .032, V = .12). More children from a high FAS background were categorized as 4+DMS (39.1%; SR = 2.11) compared to children from a moderate FAS (30.5%; SR = −.58) or low FAS (24.7%; SR = −1.62). The opposite pattern was observed for the percentages within 1-3DSS, with more children of low FAS (30.9%) in this category, compared to their peers from moderate FAS (25.7%) or high FAS (19.2%).

### Characteristics of Sport Participation as Predictors of Family Affluence

Table 2 details the results of the Ordinal Logistic Regression. Together, the predictors of single versus multi-sport and days active per week accounted for a significant amount of variance in a participant’s Family Affluence classification (the least affluent group being the top/highest category and the most affluent group being the lowest/bottom category), likelihood ratio χ^2^ (7) = 58.097, *p* < .001. Young people who participated in a single sport were 39.8% more likely to be a member of least affluent groups compared with multiple sport participants, with an odds ratio of 1.398 (95% CI, 1.200 to 1.629), Wald χ^2^ (1) = 18.502, *p* < .001. Young people who participated in fewer days of sport were more likely to come from less affluent groups; participants who played sport 2–3 days per week were 39% more likely to come from less-affluent groups compared to the more active 4+ days per week participants, with an odds ratio of 1.390 (95% CI, 1.204 to 1.605), Wald χ^2^ (1) = 20.108, *p* < .001. Boys were also 19.5% more likely to be a member of less affluent groups, with an odds ratio of 1.195 (95% CI, 1.047 to 1.363), Wald χ^2^ (1) = 7.01, *p* = .008. Age did not significantly influence the model. The interaction between the two predictor variables of Single versus Multi-Sport and Days active per week was explored within the Ordinal Logistic Regression model and not found to be significant, making no contribution to the model.

**Table 2.** Ordinal Regression Analysis on Characteristics of Sport Participation as Predictors of Family Affluence (the Least Affluent Group Being Allocated the Highest Ordinal Rank).

Parameter		B	Std. Error	95% Wald CI		Wald Chi-Square	Df	Sig	Exp(B)	95% Wald CI for Exp(B)	
				Lower	Upper					Lower	Upper
Threshold	[Family affluence = high]	−.854	.3266	−1.495	−.214	6.844	1	.009	.426	.224	.807
	[Family affluence = moderate]	1.925	.3283	1.281	2.568	34.371	1	<.001	6.855	3.602	13.046
[Gender = male]		.178	.0671	.046	.309	7.013	1	.008	1.195	1.047	1.363
[Gender = female]		0a							1		
[Single/multi sport = single sport]		.335	.0779	.182	.488	18.502	1	<.001	1.398	1.2	1.629
[Single/multi sport = multi sport]		0a							1		
[Days active in sport per week = 1]		.171	.1136	−.051	.394	2.27	1	.132	1.187	.95	1.482
[Days active in sport per week = 2–3]		.329	.0734	.185	.473	20.108	1	<.001	1.390	1.204	1.605
[Days active in sport per week = 4+]		0a							1		
Age (Scale)	1b	−.003	.025	−.052	.046	.014	1	.907	.997	.949	1.047

*Note*: a Set to zero because this parameter is redundant. CI = Confidence Interval. Sig. = *p* value. Comparisons made to Family Affluence = Low.

## Discussion

Early specialization was infrequent amongst participants in this current representative sample of Irish boys and girls. Furthermore, rates of specialization had not greatly increased by early adolescence. Thus, early specialization may not be a dominant issue within the Irish sporting context. However, children reporting high levels of family affluence were more likely to engage in multiple sports, and more likely to engage in sports 4+ days per week, relative to their peers who reported low levels of family affluence.

The low rates of early specialization and later specialization reported in the Irish dataset are inconsistent with international findings (McGowan et al., 2020; McLeod et al., 2019; Post et al., 2017; Whatman et al., 2021; Zoellner et al., 2022) in that specialization is less common in Ireland than in the U.S. and some other countries. One possible explanation for this difference relates to differences in the specialization measurement methods. The majority of previous researchers used the definition of early sport specialization proposed by Jayanthi (Jayanthi et al., 2015, 2020), where the degree of specialization was scored from 0–3 on the basis of answers to the questions: ‘Can you pick a main sport?’; ‘Have you quit other sports to focus on a main sport?’ and ‘Do you train ≥8 months in a year?’ Other studies, however, consider only the number of sports played (e.g., Bell et al., 2016; Black et al., 2019; Rugg et al., 2021), or a combination of the number of sports and months per year (Field et al., 2019). In contrast, as this study was undertaken as a secondary analysis of an existing database, we were restricted to measuring specialization on the basis of playing a single sport and training four or more times per week. As detailed above, the former criterion has been extensively used in previous research, while the latter has been included in more comprehensive definitions of specialization (Downing et al., 2020), and is consistent with the typical hours per week metric reported in other populations (Jayanthi et al., 2015; Post et al., 2017). Nonetheless, the adopted definition may be more restrictive than other definitions due to the high volume of participation specified (4+ days/week), leading to lower numbers of athletes classified as specialized.

Alternatively, the lower proportion of highly specialized individuals we observed within our Irish sample may be due to features of the national sport culture. As with rugby in Wales (Winn et al., 2017), the cultural importance of Gaelic Games and the emphasis on playing for your local community (Geary et al., 2022) mean that the incentive to sustain participation in multiple sports (i.e., Gaelic Games and another) may be higher in the Irish context than in other countries. In addition, the existence of two dominant codes of Gaelic Games, football and hurling/camogie, provides another cultural stimulus towards multi-sport participation. Other aspects of sport culture such as valuing participation in multiple sports or having traditional seasons for sports (Green & Smith, 2016) may also play a role, but these cultural factors have not been investigated within an Irish context. Irrespective of the reason why, our data suggests that early specialization in Ireland is less of a priority than elsewhere. Given the limited resources available to youth sport organizations, a more general implication of these results is that countries should not assume that issues around sport specialization are prioritized equally cross-culturally without first confirming their existence within their context.

Previous researchers identified that children whose parents hold a higher total household income are more likely to be highly specialized in sport (Jayanthi et al., 2018; Post et al., 2018, 2019, 2021b); but these findings were not replicated here. Instead, Irish children from low affluent families were more likely to participate in a single sport only. In addition, children from high affluent families were more likely to participate in sport 4+ days per week. Our results are consistent with Winn et al.’s (2017) findings that adolescent Welsh rugby players engaged on a high-performance pathway at the under-15 level had played fewer sports between the ages of 6–14 years of age if they came from more economically deprived areas. Our findings extend the relationship between socioeconomic status and specialization to the general population of sports participants. Within the context of the United States, where the majority of previous research has been conducted, parents express a strong belief that specialization increases a child’s chances of success at gaining a collegiate scholarship or professional contract (Padaki et al., 2017; Post et al., 2019, 2021b). The differing findings from our Irish sample may derive from the amateur nature of several popular Irish sports (Geary et al., 2022) and the lesser role of sports scholarships as a means of gaining access to the Irish university sector, compared to the United States. Equally, it may be that engagement in multi-sports is difficult for lower affluent families, due to their limited resources (e.g., time, money, and access to facilities) (Baker et al., 2021; Owen et al., 2022; Vella et al., 2014). For example, adolescents from medium and high affluent families identified the provision and access to local facilities as a facilitator to their physical activity, but the absence of these factors was a barrier for adolescents from low affluent families, as was the perceived lack of safety when travelling to/from these facilities (Alliott et al., 2022). Likewise, adolescents from low affluent family households were more likely to identify a lack of financial support and issues with transport as significant barriers to participating in club sport (Alliott et al., 2022), in part due to the limited control over working hours and associated “time poverty” of many of their parents (Sjödin & Roman, 2018). Additional research is required to explain the reasons for this relationship between socioeconomic status and sport participation in the Irish context.

The opportunity to engage in multiple sports during childhood and adolescence appears to provide advantages in terms of long-term skill acquisition (Arede et al., 2019; Fransen et al., 2012), the avoidance of burnout (Giusti et al., 2020) and sustained participation (Gallant et al., 2017; Murata et al., 2022; Russell, 2014). Voucher schemes have been explored as a method by which participation in community sport might be increased (Foley et al., 2021; Spence et al., 2010). For example, in Australia the Active Kids voucher programme significantly increased children’s physical activity levels, and these increases were sustained over a 6-month period (Foley et al., 2021). Likewise, Spence et al. (2010) found that a Canadian scheme which offered a voucher (in the form of a tax credit) to all families increased children’s participation in sport, particularly amongst children from the lowest income quartile. However, Spence et al. also found that parents in the lowest income quartile were significantly less aware of and less likely to claim the voucher than other income groups. This pattern of results led the authors to conclude that the outcome of the scheme was that “the rich get richer,” and that additional factors are necessary to facilitate sports participation for low socioeconomic status groups.

Encouraging multi-sport participation may be considered as a special case of retaining young people’s participation in sport in general. Rather than investing resources in developing new initiatives, it may be efficient for researchers and sports administrators to examine how initiatives designed to prevent dropout might be adapted to facilitate multi-sport participation. For example, the Keep Youngsters Involved project (a) identified 14 factors which various stakeholders can influence to prevent dropout in sport, (b) specified actions that could address these factors, and (c) provided a framework through which clubs and coaches could be encouraged to reflect upon their current practice (Murphy et al., 2018; https://tools.kenniscentrumsportenbewegen.nl/keep-youngsters-involved/hoofdstuk/research/). Three of these factors (and the associated actions) are relevant to promoting multi-sport participation amongst individuals from low socioeconomic backgrounds: Cost (e.g., give youngsters and parents the opportunity to decrease the contribution by working voluntary for the club), Accessibility (e.g., review timing and venues of training in relation to public transport), and Type of sport offer (e.g., organize exchanges between neighbouring clubs). Thus, the Keep Youngsters Involved toolkit may provide coaches and administrators with practical guidance and resources to promote multi-sport involvement amongst children and adolescents from low socioeconomic backgrounds.

### Limitations and Directions for Further Research

Acknowledging that diverse definitions of specialization exist within the literature (Bell et al., 2021; Jayanthi et al., 2020; Mosher et al., 2020), a limitation of the present study is the definition of specialization we used. While the existing data set which was exploited in this secondary analysis contained questions on the number of sports played and the number of days per week those sports were played, there were no questions relating to such other characteristics of specialization as year-round participation (Jayanthi et al., 2020) and the intensity of training (Downing et al., 2020). These additional characteristics would have been valuable to consider as a nine-year-old training in a recreational manner for six months of the year cannot be considered “specialized” to the same extent as another nine-year-old following a highly structured development programme for 12 months of the year, even when children were only playing one sport. A second limitation is that our data was drawn from participation in sport clubs (i.e., outside of school teams), due to the nature of the questions asked in the original survey. Thus, our relatively lower figures for (early) specialization are likely overestimates of the true numbers of participants who have specialized (McGowan et al., 2020). To address both limitations, in future research a more comprehensive definition of sport specialization (Downing et al., 2020) covering both schools and sport clubs could allow for a more nuanced analysis of the factors associated with performance levels (Baker et al., 2021).

## Conclusion

Neither early sport specialization nor later sport specialization were prominent within children and adolescents in Ireland. Within the Irish population, examining family affluence as a proxy measure for socioeconomic status suggests that specialization may be more commonly due to limited opportunities to engage in multiple sports, as opposed to a deliberate choice to specialize as seen in the data from the United States. When advising multi-sport participation, careful consideration should be given to whether low socioeconomic status may act as a barrier to acting upon such advice. Attention should be given to strategies that could be applied to overcome that barrier.

## Note

1. Hurling is the name given to the version of the game played by males, while Camogie is the name given to the version of the game played by females.
